# Epidemiology of extended-spectrum beta-lactamase producing Enterobacteriaceae in Qatar: A 3-year hospital-based study

**DOI:** 10.3389/frabi.2022.980686

**Published:** 2022-09-07

**Authors:** Musaed Alsamawi, Anwar I. Joudeh, Yaseer Eldeeb, Ayman Al-Dahshan, Fahmi Khan, Wissam Ghadban, Muna Almaslamani, Abdulatif Alkhal

**Affiliations:** ^1^Infectious Diseases Division, Internal Medicine Department, Al-Khor Hospital, Hamad Medical Corporation, Doha, Qatar; ^2^Internal Medicine Department, Al-Khor Hospital, Hamad Medical Corporation, Doha, Qatar; ^3^Department of Medical Education, Community Medicine Residency Program, Hamad Medical Corporation, Doha, Qatar; ^4^Internal Medicine Department, Al-Khor Hospital, Hamad Medical Corporation, Doha, Qatar; ^5^Communicable Disease Center, Hamad Medical Corporation, Doha, Qatar

**Keywords:** antibiotic resistance, community-acquired infection Enterobacteriaceae, extended-spectrum beta-lactamase, hospital-acquired infection, Enterobacteriaceae

## Abstract

**Background:**

The incidence of ESBL infections is exponentially increasing with variable prevalence among geographical areas and treatment settings. Identifying local prevalence rate and patient-related factors will help in earlier recognition and initiation of appropriate antibiotics treatment of patients with ESBL infections.

**Methods:**

Retrospective analysis of all positive cultures for ESBL producing Enterobacteriaceae collected in Al-Khor hospital from January 2010 to December 2012. ESBL bacterial isolates reported as cephalosporin-resistant or ESBL using the automated VITEK Gram-Negative Susceptibility System with cards GNS 206 and 121 were screened for ESBL detection using the disk diffusion method in keeping with the Clinical and Laboratory Standards Institute. Both descriptive and analytic statistics were applied when appropriate, and univariate analysis was used to identify significant factors.

**Results:**

Most of the ESBL-producing bacterial isolates were *E. coli*, which were also resistant to other classes of antimicrobials. Meropenem, amikacin and nitrofurantoin retained good coverage to most isolates. Klebsiella pneumonia infection was most likely associated with diabetes mellitus (*p* = 0.004), hospital-acquired infection (*p* = 0.046) and with more severe infection (*p* = 0.006). ESBL associated hospital-acquired infections were more likely to occur in older patients, those with comorbidities and with invasive device use. ESBL-associated urinary tract infections were most commonly community-acquired while ESBL associated respiratory tract infections were acquired from hospitals (*p* = < 0.001). Factors associated with mortality include treatment in the ICU (OR 104.8 [9.82–1116.96] *p* < 0.001), sepsis/septic shock (OR 20.80 (5.68–76.12) *p* < 0.001), hospital-acquired infections (OR 8.80 [1.88–41.16] *p* = 0.006) and bacteremia (OR 8.80 [1.63–47.5] *p* = 0.013).

**Conclusion:**

Multiple risk factors were associated with ESBL infections both in the community and hospital setting. Prediction tools are needed to improve the protocol of appropriate empiric antibiotic selection while preserving antimicrobial stewardship recommendations.

## Introduction

Antimicrobial resistance is one of the greatest challenges for modern healthcare. Among multidrug-resistant bacteria, the prevalence of extended-spectrum β-lactamases (ESBLs) producing Enterobacteriaceae is rising exponentially not only in hospital settings but also affecting previously healthy individuals in the community (Adler et al., 2020).

ESBLs are a group of enzymes that deactivate and provide resistance to most beta-lactam antibiotics, including penicillin, cephalosporins, and the monobactam aztreonam (Munoz-Price et al., 2019). They have been found exclusively in gram-negative organisms including Klebsiella pneumonia, Klebsiella oxytoca, Escherichia coli, Acinetobacter, Burkholderia, Citrobacter, Enterobacter, Morganella, Proteus, Pseudomonas, Salmonella, Serratia, and Shigella species (Munoz-Price et al., 2019).

Infections with ESBL-producing Enterobacteriaceae are associated with worse clinical outcomes, higher mortality rates, longer hospital stays, and greater expenses compared with similar infections with gram-negative bacteria that do not produce ESBL (Esteve-Palau et al., 2015). Furthermore, ESBL-producing Enterobacteriaceae are often resistant to other classes of antibiotics such as aminoglycosides, fluoroquinolones, and trimethoprim/sulfamethoxazole which add to their treatment difficulties (Adler et al., 2020).

Laboratory identification of ESBLs is problematic due to their heterogenicity and variable susceptibility to different oxyimino-beta-lactams. Therefore, the criteria for ESBL detection have varied over time; and clinical laboratories vary in their success in diagnosis. These diagnostic challenges interfere with ESBL identification, cause delay of appropriate antimicrobial initiation and might result in treatment failure (Munoz-Price et al., 2019; Adler et al., 2020).

Multiple risk factors are associated with this infection including recent hospital admission, residency in a long-term healthcare facility, hemodialysis, having indwelling devices, recent antibiotics use, and international travel particularly to Southern Asia (Munoz-Price et al., 2019). In addition, management and prevention of these potentially serious infections are complicated by the presence of environmental, food and animal contamination of ESBL-producing gram-negative bacteria, as well as the propensity for human to human transmission (Tal Jasper et al., 2015).

The prevalence of bacterial isolates expressing the ESBL phenotype varies across different geographical regions and it is rapidly changing, therefore, monitoring their prevalence is warranted (Tal Jasper et al., 2015; Munoz-Price et al., 2019; Adler et al., 2020). Also, it is important to identify risk factors and predictors of clinical severity in patients with ESBL-producing Enterobacteriaceae infections.

This study aims to describe the clinical characteristics and risk factors for patients with positive cultures for ESBL-producing Enterobacteriaceae causing hospital and community-acquired infections in Al-Khor hospital in Qatar and correlate these risk factors with mortality.

## Methodology

This is a retrospective cross-sectional study that included patients of all ages who had a positive culture for ESBL producing Enterobacteriaceae at Al-Khor hospital, a 115-bed general hospital in the northern region of Qatar. The study patients were identified through the database of the clinical microbiological laboratory of patients admitted to the hospital or treated in the outpatient department during the period from January 2010 to December 2012. Ethical approval to conduct this study was obtained from the Institutional Review Board at Hamad Medical Corporation (MRC reference number 13135/13). The need for consent was waived by the ethical committee as only anonymous data without patients' identifiers were provided to the research team.

ESBL bacterial isolates reported as cephalosporin-resistant or ESBL using the automated VITEK Gram-Negative Susceptibility System with cards GNS 206 and 121 (bioMérieux, Vitek Inc., Hazelwood, USA) were screened for ESBL detection using the disk diffusion method in keeping with the Clinical and Laboratory Standards Institute (CLSI) recommendation guidelines (Clinical Laboratory Standards Institute, 2010).

No sampling was done and cultures from all specimens were included (blood, sputum, tracheal aspirates, intra-abdominal collections, urine, wound cultures, surgical site infections, and skin). Patients with a second culture of similar organisms and patients with polymicrobial infections were excluded from the study.

Data obtained from the medical records were extracted electronically and manually and included: demographic data, potential risk factors for developing ESBL-producing Enterobacteriaceae, clinical management and risk factors for mortality, age, sex, type of infection, treatment in the ward, ICU, or in the outpatient department, comorbid diseases, previous antibiotic therapy, site of infection, drug susceptibility, empiric and final antimicrobial therapy, clinical outcome and inflammatory markers.

### Definitions

Previous antimicrobial therapy was recorded for those with activity against gram-negative organisms given for at least 2 days within 90 days before an episode with ESBL-producing organisms.

Hospital-acquired infection is defined as the occurrence of infection 48 h or more after hospital admission, without evidence that the infection was present or incubating on admission, in patients without prior history of stay in a healthcare facility (Friedman et al., 2002). Whereas history of hospitalization is defined as being admitted in an acute care hospital for at least 2 days in the 90 days before the sentinel infection (Friedman et al., 2002).

Colonization: which means the presence of microorganisms on skin, on mucous membranes, in open wounds, or excretions or secretions but are not causing adverse clinical signs or symptoms (Clinicians: Information about CRE|HAI|CD, 2022).

The site of infection was determined to be pneumonia, urinary tract infection, surgical site infection, intra-abdominal infection, line-related infection, and otherwise according to Centers for Disease Control and Prevention (CDC) definitions (Horan et al., 2008).

Sepsis was defined per the 2016 Society of Critical Care Medicine and the European Society of Intensive Care Medicine (SCCM/ESICM) task force as life-threatening organ dysfunction caused by a dysregulated host response to infection, while septic shock was defined as sepsis that has circulatory, cellular, and metabolic abnormalities that are associated with a greater risk of mortality than sepsis alone (Singer et al., 2016).

Empiric antibiotics were considered appropriate if the organism had *in vitro* susceptibility to at least one of the drugs administered within 72 h after the documented infection, whereas final antibiotics were considered appropriate if the organism was susceptible *in vitro* to at least one of the drugs administered after 72 h of the documented infection.

Clinical cure was defined as the resolution of signs, symptoms, and laboratory parameters that define infection for each patient. Mortality is defined as death from any cause within 6 months from the date of the first positive culture for ESBL-producing Enterobacteriaceae.

### Statistical analysis

The data were analyzed using the IBM SPSS Statistics for Windows (version 23, IBM Corp., Armonk, N.Y., USA). Both descriptive and analytic statistics were applied. For descriptive statistics, frequencies (counts) and percentages were calculated for categorical variables while means and standard deviations (SD) were calculated for contentious variables. For analytical statistics, the Fischer Exact test and Chi-Squared test were applied for categorical outcomes. Univariate analysis was used to identify significant factors, the results being presented as odds ratios (OR) with 95% confidence intervals (95% CI). Statistical significance was considered at *p* < 0.05 as a cut-off point.

## Results

**Figure 1:**
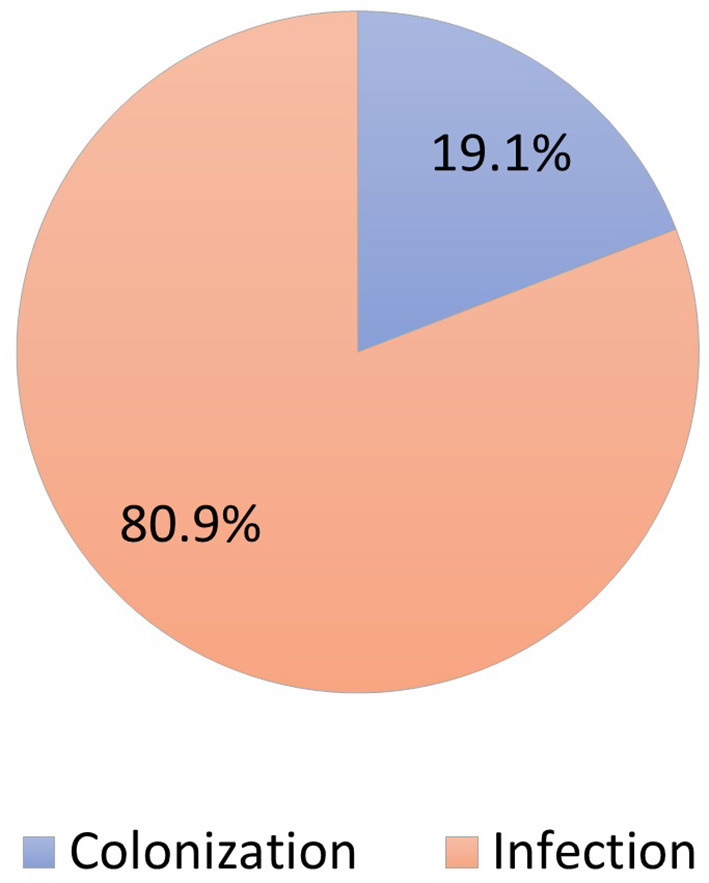
Distribution of patients colonized vs. infected with ESBL-producing Enterobacteriaceae (*N* = 230).

**Figure 2:**
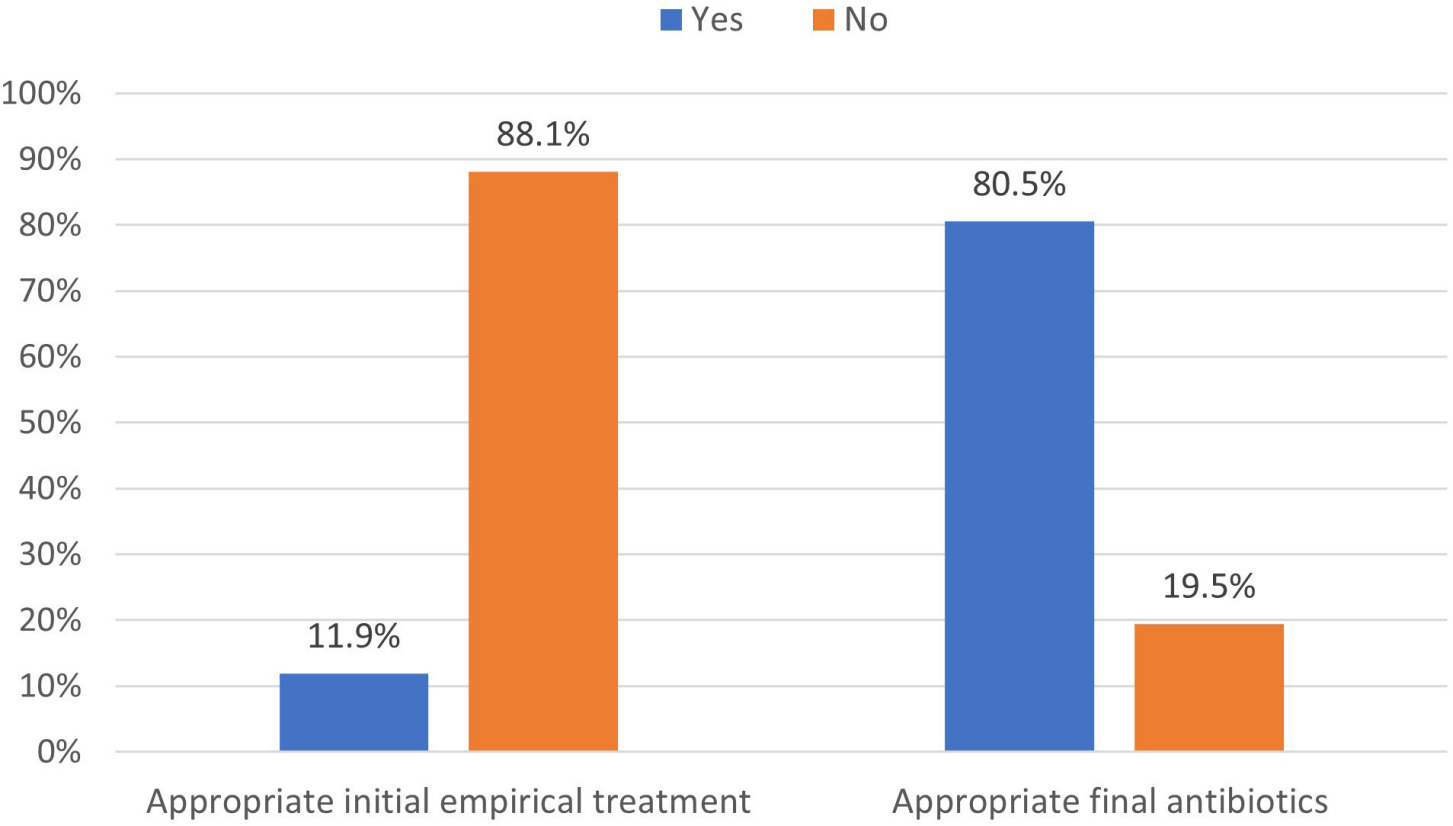
Distribution of appropriate antibiotics used in managing ESBL-producing Enterobacteriaceae (*N* = 230).

During the study period, 230 patients had culture-positive diagnosis of ESBL-producing Enterobacteriaceae. As shown in Figure 1, around 19.1% were colonized with the pathogen while the rest had clinical infection as defined by the CDC criteria (Horan et al., 2008). Only 11.9% of patients with clinical infection by ESBL-producing Enterobacteriaceae had appropriate empiric antibiotics, while 81.5% had appropriate final antibiotics treatment as shown in Figure 2.

Most of the patients had ESBL *E. coli* (*N* = 197, 85.7%), while 31 patients had ESBL K. pneumoniae (13.5%), one patient had ESBL K. oxytoca (0.4%) and one patient had ESBL P. mirabilis (0.4%). The percent resistance of E coli and K. pneumoniae against various antibiotics is depicted in Table 1.

**Table 1:** Percent resistance of Klebsiella and *E. coli* against various antibiotics (*N* = 230).

**Antibiotic name**	**E. coli resistance (%)**	**K. pneumoniae resistance (%)**
Ampicillin	100.0	100.0
Amoxi-Clavulinate	98.5	100.0
Cefuroxime	100.0	100.0
Trimethoprim-sulfamethoxazole	84.5	81.8
Ciprofloxacin	89.8	66.6
Ceftriaxone	100	100
Gentamycin	52.9	56.2
Ceftazidime	100	100
Cefepime	95.8	100.0
Nitrofurantoin	7.6	30.8
Pip-Tazobactam	23	50
Amikacin	20	0
Meropenem	0.08	0

Table 2 shows the background characteristics and risk factors of study participants. The mean age was 41.3 years ± 22.2 and just more than half were males (57.0%). Around 51% had a prior history of antibiotic use, and 55.2% had a history of hospitalization. The most common comorbidity was diabetes mellitus (27.8%) followed by chronic renal disease (9.6%). Only 8.7% of the patient had a history of invasive device insertion, and around 7.0% of the cases were complicated by sepsis or septic shock. Around two-thirds of the patient had a community-acquired infection (61.3%), and 57.4% were managed in the outpatient department. Most of the positive cultures were collected from urine (59.0%), and wound (30.6%), while positive blood and sputum cultures constituted only 7.9 and 2.6%, respectively.

**Table 2:** Background characteristics of study participants and factors associated with ESBL-producing *E. coli* and *K. pneumoniae* (*N* = 230).

**Variable**	**Total, n (%)**	***E. coli*, n (%)**	***K. pneumoniae*, n (%)**	***p*-value**
**Age (years)**				
18 or less	28 (12.2)	26 (92.9)	2 (7.1)	0.477
19–64	161 (70.0)	136 (85.0)	24 (15.0)	
65 or more	41 (17.8)	34 (82.9)	7 (17.1)	
mean ± SD	41.3 (22.2)	40.3 ± 22.3	47.6 ± 20.9	0.083
**Gender**				
Female	99 (43.0)	83 (84.7)	15 (15.3)	0.739
Male	131 (57.0)	113 (86.3)	18 (13.7)	
**History of prior antibiotic use**				
No	110 (47.8)	97 (89.0)	12 (11.0)	0.147
Yes	118 (51.3)	97 (82.2)	21 (17.8)	
**Sepsis/septic shock**				
No	214 (93.0)	186 (87.3)	27 (12.7)	0.006*
Yes	16 (7.0)	10 (62.5)	6 (37.5)	
**History of invasive device**				
No	210 (91.3)	180 (86.1)	29 (13.9)	0.502
Yes	20 (8.7)	16 (80.0)	4 (20.0)	
**History of hospitalization**				
No	103 (44.8)	87 (85.3)	15 (14.7)	0.909
Yes	127 (55.2)	109 (85.8)	18 (14.2)	
**Diabetes mellitus**				
No	166 (72.2)	148 (89.7)	17 (10.3)	0.004*
Yes	64 (27.8)	48 (75.0)	16 (25.0)	
**Chronic renal disease**				
No	207 (90.4)	179 (86.9)	27 (13.1)	0.073
Yes	22 (9.6)	16 (72.7)	6 (27.3)	
**Chronic liver disease**				
No	219 (95.2)	187 (85.8)	31 (14.2)	0.662
Yes	11 (4.8)	9 (81.8)	2 (18.2)	
**Stroke**				
No	216 (93.9)	186 (86.5)	29 (13.5)	0.125
Yes	14 (6.1)	10 (71.4)	4 (28.6)	
**Malignancy**				
No	222 (96.5)	188 (85.1)	33 (14.9)	0.606
Yes	8 (3.5)	8 (100.0)	0 (0.0)	
**Type of infection**				
Hospital-acquired	89 (38.7)	71 (79.8)	18 (20.2)	0.046*
Community-acquired	141 (61.3)	125 (89.3)	15 (10.7)	
**Department**				
Outpatient	131 (57.4)	112 (85.5)	19 (14.5)	0.197
Ward	89 (38.7)	78 (87.6)	11 (12.4)	
ICU	9 (3.9)	6 (66.7)	3 (33.3)	
**Specimen**				
Urine	135 (59.0)	119 (88.1)	16 (11.9)	0.219
Wound	69 (30.6)	58 (84.1)	11 (15.9)	
Blood	18 (7.9)	14 (77.8)	4 (22.2)	
Sputum	6 (2.6)	4 (66.7)	2 (33.3)	

Age and gender of patients with ESBL-producing *E. coli* and Klebsiella species were not significantly different. There was no difference in risk factors distribution and underlying diseases between both groups except for diabetes mellitus which was significantly more common in patients with ESBL-producing Klebsiella spp. (*p* = 0.004). In addition, infection with ESBL-producing Klebsiella spp. was significantly more associated with sepsis and/or septic shock (*p* = 0.006) and more commonly hospital-acquired (*p* = 0.046) as shown in Table 2.

Table 3 compares demographic data and risk factors of patients with hospital-acquired infection to patients with community-acquired infection of ESBL-producing Enterobacteriaceae. As shown in the table, there were statistically significant associations between older age, prior use of antibiotics, prior hospitalization, multiple underlying comorbidities (including diabetes mellitus, chronic kidney disease, chronic liver disease, stroke and malignancy) and hospital-acquired infection of ESBL-producing organisms. Moreover, most patients treated in the ICU had a hospital-acquired infection, whereas most patients treated in the outpatient department had community-acquired infection. Furthermore, sepsis and/or septic shock were significantly associated with hospital-acquired infections. Regarding the type of specimen, sputum cultures were significantly associated with hospital-acquired infection, while positive urine cultures for ESBL-producing organisms were more commonly associated with community-acquired infections.

**Table 3:** Comparison between ESBL-producing Enterobacteriaceae according to the type of infection (hospital-acquired vs. community-acquired) (*N* = 230).

**Variable**	**Community-acquired, n (%)**	**Hospital-acquired, n (%)**	***p*-value**
**Age (years)**				
18 or less	23 (82.1)	5 (17.9)	<0.001*
19–64	106 (65.8)	55 (34.2)	
65 or more	12 (29.3)	29 (70.7)	
Mean ± SD	35.6 ± 20.0	50.3 ± 22.7	
**Gender**				
Female	77 (58.8)	54 (41.2)	0.366
Male	64 (64.6)	35 (35.4)	
**History of prior antibiotic use**				
No	91 (82.7)	19 (17.3)	<0.001*
Yes	49 (41.5)	69 (58.5)	
**Sepsis/septic shock**				
No	137 (64.0)	77 (36.0)	0.002*
Yes	4 (25.0)	12 (75.0)	
**History of invasive device**				
No	136 (64.8)	74 (35.2)	<0.001*
Yes	5 (25.0)	15 (75.0)	
**History of hospitalization**				
No	77 (74.8)	26 (25.2)	<0.001*
Yes	64 (50.4)	63 (49.6)	
**Diabetes mellitus**				
No	121 (72.9)	45 (27.1)	<0.001*
Yes	20 (31.3)	44 (68.8)	
**Chronic renal disease**				
No	137 (66.2)	70 (33.8)	<0.001*
Yes	4 (18.2)	18 (81.8)	
**Chronic liver disease**				
No	139 (63.5)	80 (36.5)	0.003
Yes	2 (18.2)	9 (81.8)	
**Stroke**				
No	139 (64.4)	77 (35.6)	<0.001*
Yes	2 (14.3)	12 (85.7)	
**Malignancy**				
No	139 (62.6)	83 (37.4)	0.032
Yes	2 (25.0)	6 (75.0)	
**Department**				
Outpatient	94 (71.2)	38 (28.8)	<0.001*
Ward	45 (50.6)	44 (49.4)	
ICU	2 (22.2)	7 (77.8)	
**Specimen**				
Urine	102 (75.6)	33 (24.4)	<0.001*
Wound	29 (41.4)	41 (58.6)	
Blood	9 (50.0)	9 (50.0)	
Sputum	0 (00.0)	6 (100.0)	

Multiple risk factors were significantly associated with mortality in patients with ESBL-producing Enterobacteriaceae as shown in Table 4. Patients with chronic liver disease and malignancy had the highest risk for mortality (OR 15.07 [95% CI 3.65–62.16] *p* = 0.001, and OR 14.20 [95% CI 2.93–68.88] *p* = 0.005, respectively), whereas patients with diabetes mellitus had OR of 5.79 [95% CI 1.68–19.96] *p* = 0.002. Other risk factors identified included recent hospitalization (OR 9.67 [95% CI 1.22–76.22] *p* = 0.009) and invasive device use (OR 14.57 [95%CI 4.16–51.10] *p* < 0.001). In addition, hospital-acquired infections, and positive blood cultures for ESBL-producing Enterobacteriaceae were associated with higher risks of mortality with an OR of 8.8 for each with statistically significant *p*-values. Moreover, treatment in the ICU was associated with the highest OR among all other risk factors with an OR of 104.8 (9.82–1116.96) *p* < 0.001.

**Table 4:** Comparison of participants characteristics and the outcome (*N* = 230).

**Variable**	**Died, n (%)**	**Survived, n (%)**	**Odds ratio (95% CI)**	***p*-value**
**Organism**				
K. pneumoniae	3 (9.1)	30 (90.9)	2.08 (0.53, 8.15)	0.283
*E. coli*	9 (4.6)	187 (95.4)	1	
**Type of infection**				
Hospital-acquired	10 (11.2)	79 (88.8)	8.80 (1.88–41.16)	0.006*
Community-acquired	2 (1.4)	139 (98.6)	1	
**Department**				
Outpatient	1 (0.8)	131 (99.2)	1	0.025*
Ward	7 (7.9)	82 (92.1)	11.2 (1.35–92.55)	<0.001*
ICU	4 (44.4)	5 (55.6)	104.8 (9.82–1116.96)	
**Specimen**				
Urine	3 (2.2)	132 (97.8)	1	0.102
Wound	5 (7.1)	65 (92.9)	3.39 (0.79–14.60)	0.013*
Blood	3 (16.7)	15 (83.3)	8.80 (1.63–47.5)	0.080
Sputum	1 (16.7)	5 (83.3)	8.80 (0.77–100.25)	
**Sepsis/septic shock**				
Yes	6 (37.5)	10 (62.5)	20.80 (5.68–76.12)	<0.001*
No	6 (2.8)	208 (97.2)	1	
**History of invasive device**				
Yes	6 (30.0)	14 (70.0)	14.57 (4.16–51.10)	<0.001*
No	6 (2.9)	204 (97.1)	1	
**History of hospitalization**				
Yes	11 (8.7)	116 (91.3)	9.67 (1.22–76.22)	0.009*
No	1 (1.0)	102 (99.0)	1	
**Diabetes mellitus**				
Yes	8 (12.5)	56 (87.5)	5.79 (1.68–19.96)	0.002*
No	4 (2.4)	162 (97.6)	1	
**Chronic renal disease**				
Yes	6 (27.3)	16 (72.7)	12.56 (3.63–43.45)	<0.001*
No	6 (2.9)	201 (97.1)	1	
**Chronic liver disease**				
Yes	4 (36.4)	7 (63.6)	15.07 (3.65–62.16)	0.001*
No	8 (3.7)	211 (96.3)	1	
**Stroke**				
Yes	4 (28.6)	10 (71.4)	10.40 (2.68–40.43)	<0.001*
No	8 (3.7)	208 (96.3)	1	
**Malignancy**				
Yes	3 (37.5)	5 (62.5)	14.20 (2.93–68.88)	0.005*
No	9 (4.1)	213 (95.9)	1	

## Discussion

In this study, we report demographic data and clinical risk factors for ESBL-producing Enterobacteriaceae in 230 patients treated in Al Khor hospital during a 3-year period. *E. coli* and Klebsiella pneumonia constituted most of the organisms producing ESBL enzymes, with only 12% of patients receiving appropriate empiric antibiotics therapy. We identified multiple risk factors for ESBL-associated hospital-acquired infections as well as mortality.

Rising resistance among the most common human pathogen Enterobacteriaceae is alarming due to its associated burden on both patients and public health. ESBL-producing Enterobacteriaceae isolates do not only convey resistance to beta-lactam antibiotics but also frequently exhibit cross-resistance to other antimicrobial classes. Delay in appropriate antibiotic administration is associated with worse clinical outcomes, a longer length of hospital stay and higher health-related costs (Lee et al., 2006). Almost all bacterial isolates in the present study were sensitive to carbapenems which are in support of the role of carbapenems as the mainstay of therapy for severe ESBL infections (Rodríguez-Baño et al., 2018; Adler et al., 2020). In addition, amikacin showed potent *in-vitro* antibacterial activity in this study, which was consistent with other study findings (Rodríguez-Baño et al., 2018), however, its use is limited by its potential renal toxicity. Unfortunately, oral therapeutics against ESBL-producing organisms are limited. In the current study, resistance rates against trimethoprim-sulfamethoxazole and ciprofloxacin were all high. Similar findings were reported in a prospective multi-center observational study conducted in the United States of America where only 11% and 32% of community-acquired ESBL-producing E coli isolates were susceptible to fluoroquinolones and trimethoprim-sulfamethoxazole, respectively (Doi et al., 2013). However, nitrofurantoin resistance remains relatively rare especially among ESBL-producing *E. coli* despite several years of large use which provides a reasonable option for oral treatment of uncomplicated infections in the outpatients.

Klebsiella pneumoniae and *Escherichia coli* are the two major ESBL-producing organisms isolated worldwide (Pitout and Laupland, 2008). In the current study, most of the ESBL-producing bacterial isolates were *E. coli*. However, ESBL- producing Klebsiella pneumonia was more statistically associated with diabetes mellitus, hospital acquired infections and sepsis. The propensity of ESBL-producing Klebsiella pneumonia to cause hospital-acquired infection has been well-recognized since the 1980's, whereas later in the 2000's, *E. coli* has emerged as an important source for community-onset ESBL- associated infections (Pitout et al., 2005; Pitout, 2010). According to Vading et al., impaired host defense place patients at greater risk of developing invasive infection by Klebsiella pneumoniae compared to *E. coli*. The authors analyzed risk factors and prognosis of invasive infections caused by Klebsiella pneumonia vs. *E. coli* in 599 patients and found that Klebsiella pneumonia was associated with higher levels of comorbidities as well as 90-day mortality in comparison to invasive *E. coli* infections (Vading et al., 2018). Therefore, empiric treatment for Klebsiella pneumonia should be taken into consideration when managing severely ill patients with comorbidities including diabetes and in patients with nosocomial infections.

In the hospital settings, we identified several risk factors that were significantly associated with ESBL infections including infection by Klebsiella pneumonia, recent antibiotics use, use of invasive device, treatment in the intensive care unit (ICU), more severe illness and respiratory tract infections. These findings were also consistent with Pitout and Laupland conclusions on the predictors of hospital vs. community-onset infections caused by ESBL-producing bacteria (Pitout and Laupland, 2008). This highlights the importance of following appropriate infection control measures and antibiotics management strategies in hospitals in order to reduce the burden of hospital-acquired ESBL infections.

ESBL infection-associated mortality is difficult to interpret in the presence of multiple confounders. In the present study, only 11% of patients received appropriate empiric antibiotic therapy within 72 h and multiple factors were associated with higher odds for mortality including hospital-acquired infections, presence of sepsis/septic shock, treatment in the ICU, use of an invasive device, positive blood culture and the presence of comorbidities. Similarly, Park and his colleagues studied clinical and microbiologic features of 300 cases of ESBL-producing *E. coli* at three medical centers in the United States. The authors found that predictors of 28-day mortality included dialysis in the past 90 days (OR, 6.65; 95% CI: 1.85–23.84; *P* = 0.004), the presence of a vascular catheter at enrollment (OR, 5.21; 95% CI: 1.69–16.02; *P* = 0.004), hospital-acquired infection (OR, 5.21; 95% CI: 1.69–16.02; *P* = 0.004) and liver disease (OR, 4.52; 95% CI: 1.44–14.23; *P* = 0.01). On the other hand, predictors of survival at 28-day included favorable baseline health status (OR, 0.39; 95% CI: 0.16–0.95), and appropriate empirical antimicrobial therapy given in the first 72 h (OR, 0.42; 95% CI: 0.20–0.88) (Park et al., 2012).

The isolated impact of ESBL infections on mortality was analyzed in a meta-analysis on 16 studies conducted from 1996 to 2003 which found a significant delay in appropriate antibiotics therapy (RR 5.56, *p* < 0.001) and a significant rise in mortality among patients with ESBL-associated bloodstream infections (RR 1.85, *p* < 0.001) (Schwaber and Carmeli, 2007). As the delay in appropriate antibiotics therapy in the strongest modifiable independent predictor for mortality in sepsis (Paul et al., 2010), this might be one of the main reasons for worse clinical outcomes among patients with ESBL infections (Schwaber and Carmeli, 2007). Therefore, identifying risk factors for ESBL infections in both hospital and community settings, early pathogen detection, and standardization of appropriate antibiotics administration based on reliable prediction tools could shorten the time to initiation of appropriate antimicrobial therapy and improve patient outcomes.

This study has several limitations. Due to the retrospective nature of data collection, we could not control all variables and some of the medical records were not complete. A follow-up longitudinal study will be more helpful in establishing causal relationships between risk factors and outcomes. Also, we did not use an objective assessment tool to assess severity at the onset of infection that would probably affect the selection of empiric antibiotics and treatment outcome. Lastly, we could not provide information on genotypic analysis of ESBL-producing bacterial isolates in our study.

## Conclusion

We identified multiple risk factors associated with ESBL infections both in the community and hospital setting. In the setting of increased prevalence of antibiotics resistance, identifying risk factors associated with infections by ESBL-producing bacteria may improve the protocol of empiric antibiotic treatment while preserving antimicrobial stewardship recommendations. Further research is needed to improve prediction tools for ESBL infections for both hospital as well as community-acquired infections. In addition, investigational efforts should be directed toward infection control measures, early pathogen identification and antibiotics stewardship programs.

## Data availability statement

The original contributions presented in the study are included in the article/supplementary material, further inquiries can be directed to the corresponding author/s.

## Ethics statement

The studies involving human participants were reviewed and approved by the Institutional Review Board at Hamad Medical Corporation (MRC reference number 13135/13). Written informed consent from the participants' legal guardian/next of kin was not required to participate in this study in accordance with the national legislation and the institutional requirements.

## Author contributions

MusA: conceptualization, methodology, validation, investigation, data curation, writing–original draft, writing–review and editing, and project administration. AJ: conceptualization, resources, writing–original draft, writing–review & editing, and visualization. YE: conceptualization, methodology, and supervision. AA-D: validation, formal analysis, and visualization. FK: resources, conceptualization, methodology, and validation. WG: writing–review & editing, supervision, and project administration. MunA and AA: writing–review & editing, supervision, and project administration. All authors contributed to the article and approved the submitted version.

## Funding

The publication of this article was funded by Qatar National Library.

## Conflict of interest

The authors declare that the research was conducted in the absence of any commercial or financial relationships that could be construed as a potential conflict of interest.

## Publisher's note

All claims expressed in this article are solely those of the authors and do not necessarily represent those of their affiliated organizations, or those of the publisher, the editors and the reviewers. Any product that may be evaluated in this article, or claim that may be made by its manufacturer, is not guaranteed or endorsed by the publisher.
